# Printing Polymeric Convex Lenses to Boost the Sensitivity of a Graphene-Based UV Sensor

**DOI:** 10.3390/polym14153204

**Published:** 2022-08-05

**Authors:** Jonghyun Kim, Dongwoon Shin, Jiyoung Chang

**Affiliations:** 1 Department of Mechanical Engineering, Keimyung University, 1095 Dalgubeol Daero, Daegu 42601, Korea; 2 Max Planck Institute for Polymer Research, Ackermannweg 10, 55128 Mainz, Germany; 3 Department of Mechanical Engineering, University of Utah, Salt Lake City, UT 84112, USA

**Keywords:** printing, electrospinning, polymer, lens, graphene, sensor, fabrication, manufacturing, sensitivity

## Abstract

Ultraviolet (UV) is widely used in daily life as well as in industrial manufacturing. In this study, a single-step postprocess to improve the sensitivity of a graphene-based UV sensor is studied. We leverage the advantage of electric-field-assisted on-demand printing, which is simply applicable for mounting functional polymers onto various structures. Here, the facile printing process creates optical plano-convex geometry by accelerating and colliding a highly viscous droplet on a micropatterned graphene channel. The printed transparent lens refracts UV rays. The concentrated UV photon energy from a wide field of view enhances the photodesorption of electron-hole pairs between the lens and the graphene sensor channel, which is coupled with a large change in resistance. As a result, the one-step post-treatment has about a 4× higher sensitivity compared to bare sensors without the lenses. We verify the applicability of printing and the boosting mechanism by variation of lens dimensions, a series of UV exposure tests, and optical simulation. Moreover, the method contributes to UV sensing in acute angle or low irradiation. In addition, the catalytic lens provides about a 9× higher recovery rate, where water molecules inside the PEI lens deliver fast reassembly of the electron-hole pairs. The presented method with an ultimately simple fabrication step is expected to be applied to academic research and prototyping, including optoelectronic sensors, energy devices, and advanced manufacturing processes.

## 1. Introduction

Ultraviolet (UV) is widely used in daily life as well as in industrial manufacturing, such as in virus sterilization, insect removal, aesthetic curing, lighting for biotherapy, and bonding for structural assembly. However, excessive use of UV can cause serious damage to integrated devices and even to users. Hence, precise measurement is greatly important to properly exploit the advantages of UV [1,2,3]. Among measurement methods, the use of graphene (GP) for UV sensing has received great attention for its lightweight, superior electrical and mechanical properties [4,5,6], chemo-functionality [7,8], and quick opto-response [9,10]. To overcome the sensor’s aboriginal capabilities, various techniques to improve sensitivity have recently been suggested. First, utilizing nanostructures for modulation of response was reported, but the methods involve time-consuming fabrication processes, expensive facilities, or are infeasible for microscale devices [11,12,13]. To avoid complicated structures, a surface modification of graphene using specialized functional molecules was suggested. However, such a method inevitably involves expensive materials and treatment processes [14]. Next, introducing high humidity in the sensing sequence can be one of the alternates for enhancing sensitivity as it is simple, cost-efficient, and applicable to microscale devices. However, exposure to graphene in a highly humid atmosphere for a long time is known to degrade photosensitivity [15]. Furthermore, it was reported that the generation of a graphene oxide layer underwater improves the sensitivity, yet in turn, the use underwater sacrifices the mobility of compact electronic devices [16]. Likewise, Li [17], Hernaez [18], and Sanctis et al. [19] presented comparative performance tables in reviews of graphene-based photodetectors, which supported insightful background studies. As emerging materials and manufacturing technologies such as graphene synthesis and printings have been developed, there is room for improving the performance of the sensor. Thus, it is necessary to develop a new strategy for UV sensing that promises superior sensitivity by combining low-cost materials, simple preparation, easy fabrication, and harmonization with the graphene.

The plano-convex lens has been utilized in a wide range of applications by using its well-established optical theory, including as a solar concentrator in photovoltaic systems [20], as optical sensors in automotive parts [21], in laser exposure systems [22], and as an artificial eye in biomimicry robotics [23]. The simplification of the fabrication process is important from a productive point of view. To date, a variety of fabrication processes have been suggested to create plano-convex lenses, including inkjet printing [24], molding [25], and reflowing [26]. However, they possess complicated steps such as the need for a customized needle and actuator [24], inevitable part replacement [25], and the difficulty of dimension control of the individual products [26]. Besides, dimensional controllability of the lens is the foremost interest. In particular, adjusting the focal point in the process is a fastidious task. Even though several techniques, such as molding and etching [27], have been demonstrated, these techniques are not capable of obtaining a smooth curved surface. Similar to our method, one method used the electrowetting of tuned contact angles of the individual polymeric lens by employing an external electric field (EF) after shooting the droplet [28]. However, the process has a critical issue where the strong EF can damage the graphene layer. Since surface treatments also have widely been studied [29,30], the processes are limited in the selection of substrate and are not appropriate for large manufacturing as each lens needs to be treated separately.

**Figure 1:**
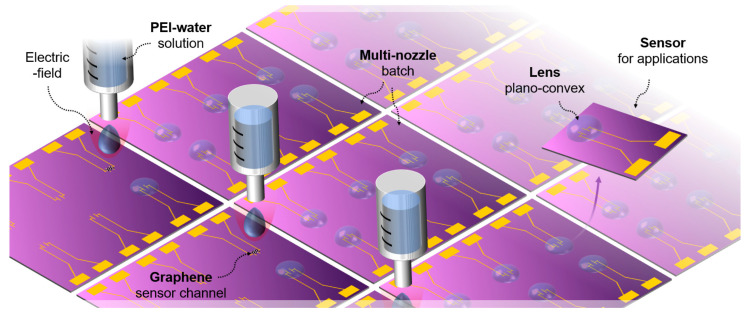
Concept of electric-field-assisted printing of PEI–water solution: the multi-nozzle provides high-throughput batch fabrication of plano-convex lenses to improve the sensitivity of graphene-based UV sensors. The sensor can be applied to various applications.

As shown in Figure 1, here, we employ a simple printing method to create a functional lens on a UV sensor, leveraging the advantage of the EF. In order to use printing as the core method of UV sensor manufacturing, we looked at the types of printing categorized into three major technologies, i.e., stereolithography (focused laser curing), deposition printing (extrusion) [31], and EF-assisted printing [32]. In stereolithography, layered base molds are formed by photopolymerization. The resin stored in the tank selectively is exposed to light. Since the photopolymerized product is submerged in the resin, this method allows lower design freedom than constrained surface printings for the lens mounting [33]. Moreover, the photopolymers and the tools used in stereolithography typically are expensive, thus it is not a cost-effective method compared to deposition printing [34]. On the other hand, deposition printing is a facile process that uses thermoplastic filaments or photopolymers. Recently, fused deposition printing has become the most common technique for producing thermoplastic filaments. Easily accessible materials, such as acrylonitrile butadiene styrene (ABS) [35], polylactic acid (PLA), and thermoplastic polyurethane (TPU), can be extruded rapidly. However, since its ability to fuse with adjacent layers is low, fused deposition is inappropriate for use with sensor devices that require controllable high-gloss curvature [36]. The EF-assisted printing can shoot a highly viscous polymer droplet and create the optical plano-convex lens on a sensor surface. The fundamental idea behind this technique is based on near-field electrospinning (NFES) [37], a nanofiber-producing method utilizing electrohydrodynamic behavior in which the polymeric jet flow rate is closed related to the amplitude of the EF. In our prior work, electrospinning was initiated when the EF between the needle tip and the collector exceeded the threshold level, leading to the electrostatic force acting between the ion inside the polymer solution and the conductive collector being stronger than the surface tension of the polymer [38]. However, when an excessive level of the EF was formed between the tip and the collector, the unbalanced EF created a discontinuous polymer jet rather than forming a continuous fiber [37]. When the pulling force by both the electrostatic force and gravity on the droplet exceeded the capillary force, the droplet was ejected and fell toward the device [39,40]. In a recent publication [32], we presented the printing system for a macroscale lens array which was integrated into a microscope as a teleconverter, into fluidic tubing as a magnifier, into a laser exposing as a concentrator, and into an LED bulb encapsulating as a light diffuser. By leveraging this interesting phenomenon, we can apply this principle to the formation of a plano-convex lens by accelerating and colliding droplets onto the graphene-based UV sensor channel.

As a result, the EF-printing process fabricates functional lenses that can significantly improve the sensitivity of graphene-based UV sensors. We first synthesized a graphene layer and transferred it onto a microdevice. The EF printing draws a microfiber etch-block that can pattern microscale graphene sensor channels. Batch fabrication using a multi-nozzle with adjusting individual EF modulation accelerates, resulting in continuous variation of the focusing power of lenses, which provides an affordable and reliable fabrication, time-efficient, and low-cost process. Accordingly, optically concentrated UV rays that come through the refractive polymeric convex lens provide enhanced photon energy onto the graphene sensor channel. The UV sensing response is outstandingly boosted compared to graphene sensors without lenses, which is coupled with the change in resistance (∆R) and which is proportional to UV photons. This result provides enhancement of small form-factor optoelectronic sensors through single-step postprocessing. It is envisioned that the method can be leveraged for applications in academic research and prototyping, including optoelectronic sensors, energy devices, and advanced manufacturing processes.

## 2. Materials and Methods

### 2.1. Materials

**Figure 2:**
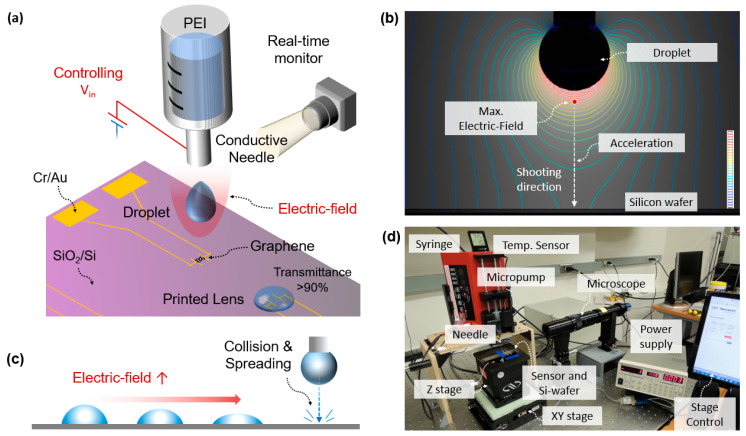
(**a**) Electric-field (EF)-assisted droplet printing mode and printed lens on the graphene-based sensor surface. (**b**) Simulated EF distribution, where the strongest EF is expected at the lowest point of the droplet. The droplet flies on a pathway where the strongest electrostatic force is generated. (**c**) Schematic of droplet variation. The lenses are fabricated with different curvatures by varying the applied voltage. The stronger the EF, the larger the collision and spreading. (**d**) EF-assisted on-demand printing system.

Figure 2a provides the materials used for the sensor device, which are listed in the order of the fabrication process. For the sensor substrate, silicon dioxide (300 nm, SiO_2_), was grown on a silicon wafer. Chrome (Cr) and gold (Au) for electrodes (purity 9.999%) were sequentially patterned by sputtering and mask-based UV photolithography. The graphene sheet was grown using a copper (Cu) foil. Cu was etched with 2% ammonium persulfate. The graphene was transferred using poly(methyl methacrylate) (PMMA). Raman spectroscopy and a scanning electron microscope (SEM, Helios Nanolab 650, FEI) were employed for graphene analysis. Polyethylene oxide (PEO, powder, Mw 4,000,000, Sigma-Aldrich) was diluted in DI water (2 wt%). The transparent polyethyleneimine (PEI) solution for the lens was purchased from Sigma-Aldrich (Mw: 600,000–900,000, dissolved in 50% H_2_O by manufacturer, transmittance >90%, hydrophilic, quality level 200, viscosity 18–40 Pas at 20 degrees, refractive index 1.44–1.45). 

### 2.2. EF-Printing Technology

Figure 2b provides a simulation analysis using a COMSOL electrostatic module to verify the effect of the EF. The strongest electrostatic force is generated at the lowest point of the droplet. The droplet can be shot and accelerated through a pathway where the strongest EF is prompted. The stronger electrostatic force associated with the higher applied voltage resulted in a faster droplet and stronger collision on the device, as shown in Figure 2c. Even with droplets of the same size, the level of the EF can be utilized to control the dimensions of the plano-convex lenses, including the radius of curvature, diameter, and thickness associated with focal length, effectively [32].

### 2.3. Printing System

Figure 2d shows an in-house built printing system that can precisely deposit polymeric droplets on the surface of the device [32]. The printing system consisted of a micropump (New Era Pump Systems), a syringe, a metallic needle (Nordson, OH), an XYZ motorized moving stage (ONE-XY100 for the XY-axis, origin repeatability ± 0.1 μm, minimum incremental motion 0.05 μm; GTS30V for the Z-axis, origin repeatability ± 0.05 μm, minimum incremental motion 0.1 μm; Newport, Irvine, CA, USA), a silicon wafer, a power supply (PS350, Stanford Research System^®^), a long-distance microscope (K2 DistaMax, Infinity), a USB camera (Basler ace), a hygrometer, and a thermometer. A UV flashlight (TaoTronics, Shenzhen, China) with a wavelength of 365 nm was used for exposure. A digital optical photometer (2835-C, Newport, Irvine, CA, USA) and probe (818-SL, Newport, Irvine, CA, USA) measured UV irradiance. The temperature sensor maintained a temperature of 70 °F and a humidity of 20%. A self-written LabVIEW graphical user interface (GUI) system monitored three camera modules in real time and precisely controlled the XYZ motorized stage. The heavily doped silicon wafer was mounted on the XYZ stage and utilized as a conductive target collector. System parameters such as applied pressure, the hydrophobicity of the needle [41], and the inner/outer diameter of the needle [42] played effective roles in determining the size of the droplet. The droplet printing system can simply transform into NFES by reducing the tip-to-collector distance and the applied voltage, as described previously in the introduction.

### 2.4. Fabrication Processes

**Figure 3:**
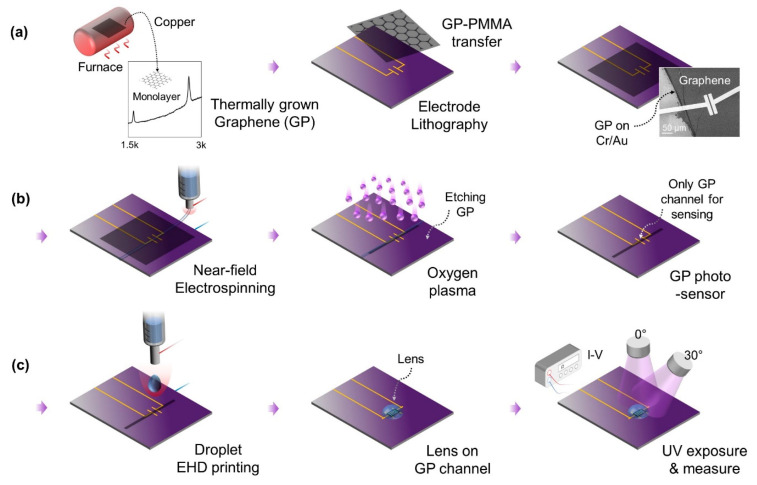
Fabrication of graphene-based photosensor. (**a**) Synthesis of single-layer graphene and transferring on the sensor. Raman spectroscopy verifies the graphene. Cr/Au electrodes are patterned by employing UV photolithography. (**b**) Facile patterning of a graphene channel via near-field electrospinning. PEO is spun on the graphene surface. Oxygen plasma etches the graphene. The protected region covered by the spun fiber remains as a graphene sensor channel (**c**) for droplet printing and measurement. The droplet is mounted on the graphene channel. UV exposure is from 0 and 30 degrees. Change in resistance (∆R) is proportional to UV photon.

Figure 3 shows the fabrication process of the graphene-based photosensor with a lens, which includes three key fabrication groups: (a) a graphene synthesis and transfer onto a sensor chip, (b) a formation of the graphene channel using NFES, and (c) the formation of the lens using droplet printing.

#### 2.4.1. Graphene Synthesis

Details of graphene synthesis and microscale channeling can be found in previous works, which provide originality and in-depth information [43,44]. In Figure 3a, the single-layer graphene was synthesized by the chemical vapor deposition (CVD) process [43,44]. A copper film was used for the growth substrate. The film was exposed to H_2_ for 100 sccm for 1 min and CH_4_ for 30 sccm for 3 min in the furnace at 550 °C. Then, 10 sccm of H_2_ flowed for 100 min in the 1050 °C furnace. After a preheating process, the film was exposed to both H_2_ of 10 sccm and CH_4_ of 22 sccm for 85 min simultaneously. The furnace was shut off after 25 min into the growth process. All gases were stopped after 85 min. The film was stored in the furnace for natural cooling to room temperature overnight. In the subset plot, the grown layer was identified by matching the characteristic wavelength of the single-layer graphene-based on the characterization by Raman spectroscopy [45].

#### 2.4.2. Graphene-PMMA Transferring

The synthesized graphene sheet was then transferred onto the device surface by a poly(methyl methacrylate) (PMMA) transfer technique [44]. The PMMA was spin-coated on the synthesized graphene in the copper film. The baked graphene-PMMA-Cu sheet was floated on a 2% ammonium persulfate solution to etch the Cu layer. After 6 h, the graphene-PMMA sheet was scooped up and transferred onto the device. The graphene-PMMA sheet was naturally dried at room temperature for 24 h. Then, the PMMA layer on the top surface was removed by putting it in acetone for 6 h. The subset SEM image shows that the graphene sheet, as a photoabsorber, was successfully mounted on the device. Conductive electrodes and contact pads were patterned using UV photolithography and Cr/Au sputtering on the SiO_2_-deposited wafer.

#### 2.4.3. Graphene Channel via Near-Field Electrospinning

Figure 3b follows a facile patterning of the graphene channel using a fiber etch-block. We used the printing system in NFES mode to deposit microfibers by setting the process parameters to 0.8 kV of the applied voltage and 1.0 mm of the distance between needle and substrate. To deposit the straight-line fiber, the jet impact speed and the collector speed should be matched [46]. We used an image processing method to account for the jet impact speed [37], then the collector speed was equalized to the estimated jet impact speed. Since an ultrahigh molecular weight of PEO is used as a rich crystalline polymer solution for the NFES, the deposited fiber (width of 80–100 µm) served as a barricade mask that endures the oxygen plasma while etching the graphene. As a result, only the protected region covered by the spun fiber remained as a graphene photosensor channel [43,47], whereas the uncovered graphene was fully eliminated [48]. 

#### 2.4.4. Electric-Field-Assisted Lens Printing

Figure 3c and the following sections provide the details of the droplet printing highlighted in this paper. At the beginning of the droplet printing process, the micropump pushes out the polymer solution through the needle, forming a droplet via the balance between the capillary force and the gravitational force. Once the desired droplet size is formed at the end of the needle, the micropump is turned off and the EF is generated by applying an electrical potential difference between the needle and the silicon wafer. The silicon wafer and the metal needle tip are connected to a high voltage supply to provide an electric potential to generate the EF (EF = applied voltage/distance between the needle and substrate). The programmable XYZ stage is used to locate the position of the droplets and to adjust the tip-to-collector distance. It was reported that electric discharge occurs when a high EF is applied with a short tip-to-collector distance [46]. However, in our design, the SiO_2_ is chosen for the dielectric surface layer of the sensor device as SiO_2_ not only allows the deposition of plano-convex lenses owing to its hydrophobicity, but also prevents the electrical discharge in the EF-assisted printing process.

Here, PEI hydrogel was chosen as the key material of the plano-convex microlenses. The PEI solution has an appropriate high viscosity (18–40 Pa s) to form a low-aspect ratio semi-ellipse convex lens. Additionally, the PEI solution has advantageous optical transmittance (above 90%) and a refractive index of 1.45 in a wide range of wavelengths, which are crucial factors in determining optical performance. The PEI lenses with the diameter of the submillimeter are fabricated by use of a microneedle (32 gauge, 235 µm of outer diameter, 108 µm of inner diameter). With precise control, the printing system deposits the microlens on the graphene channel. The digital optical photometer and probe support controlling the UV irradiance, and the I-V curve is measured in real time.

#### 2.4.5. Controlling Curvature of Lenses

In this work, we demonstrate the consistency of the droplet size by controlling the flow with the EF and vision monitoring. The dimensions of the lenses vary the optical refraction, which affects the boosting effect of the UV sensor. The ability to precisely control the lens dimensions expands the stable manufacturability and selectivity of the sensor’s measuring range. According to a relationship between the f-number and aperture, focal point = f-number × aperture, where the focal point is related to the diameter of the lens, which is controlled via corresponding droplet volume and EF [32]. The radius of the curvature of the lens significantly depends on the kinetic energy of the droplet and liquid–surface interaction upon collision on the substrate [49]. Hence, the radius of curvature of the droplet can be effectively adjusted by controlling the applied voltage. Therefore, a higher EF leads to more kinetic energy of the droplet in the fixed distance between the needle and the target surface, resulting in a stronger collision and spreading of the droplet. We fixed the distance between the needle and substrate distance to be 3 mm while the applied voltage was adjusted between 1.5 and 3.5 kilovolts (kV) to create the various curvatures of the lenses.

### 2.5. Principle of Boosting Sensitivity

**Figure 4:**
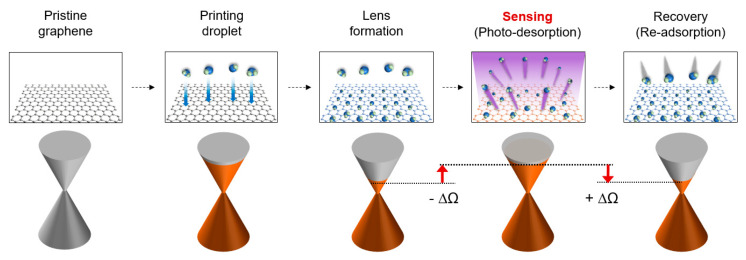
Boosting mechanism in UV measurement. UV sensing and recovery sequence of functionalized graphene. Upon UV exposure, the photon energy excites electron-hole pairs, increasing the resistance (Ω). Without UV exposure, molecules are readsorbed on the graphene layer, resulting in recovery, in which the printed polymeric lens performs boosted sensing and recovery in large ± ∆R.

As shown in Figure 4, a photodesorption and a readsorption of molecules contained in the PEI–water droplet are keys to boosting the sensitivity of the photosensing. Although the ideal graphene has a pristine phase, the in-house-synthesized graphene exhibits a *p*-type phase due to the polymer residues and O_2_ introduced during the device fabrication and transfer sequence [50,51]. In general, once H_2_O molecules from the air are adsorbed on the graphene surface [51], the Fermi level lowers the Dirac point and the resistance (R). Upon UV exposure, R (Ω) increases. The photon energy excites the electron-hole pairs (n), which can be expressed as molecular photodesorption (dn/dt = photon-flux × number of molecules × photodesorption cross-section) [52]. The photodesorption, which is coupled with the change in resistance (∆R), is proportional to both UV photons and the number of adsorbed molecules. The sensitivity is calculated by the relative resistance change, ∆R R_0_^−1^, where R_0_ is the initial resistance of a graphene channel. Without UV exposure, H_2_O molecules readsorbed on the graphene layer modulate the local carrier concentration, resulting in the recuperation of ∆R R_0_^−1^ [51]. 

## 3. Results and Discussion

### 3.1. Lens Fabrication and Products

**Figure 5:**
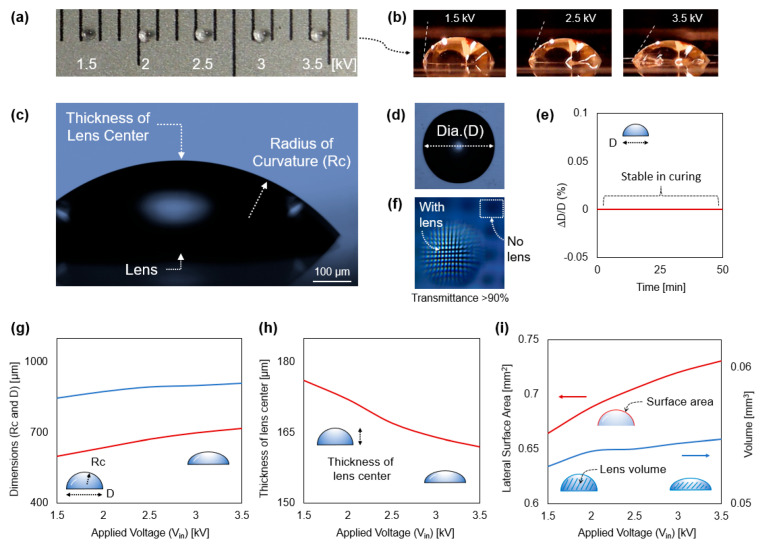
Products and quantification. (**a**) Droplets printed by V_in_ from 1.5 to 3.5 kV with 0.5 kV increment. (**b**) The larger the V_in_, the smaller the static contact angle and the larger the radius of curvature. (**c**,**d**) The side and top microscopic view of the lens. (**e**) The diameter (aperture) of the lens is maintained during printing and solidification. (**f**) Imaging mode of the printed droplet. The droplet printed on the transparent substrate magnifies wire grids through the refraction. (**g**) The higher V_in_ causes the larger diameter and the larger radius of curvature. (**h**) The higher V_in_ leads to the smaller the thickness of the lens center. (**i**) The higher V_in_ produces large lateral surface area. The volume of the droplet is maintained through the vision control system.

Figure 5a introduces sequentially varied lens products for a comparison of dimensions. To verify the dimensional stability of lens products of the droplet printing system, the droplet was directly printed on a transparent film substrate (refractive index 1.57, transmittance 91%, Thermanox^®^). The printing process did not exhibit noticeable differences in micrometers with or without the presence of the thin film. The effect of placing the thin film (ε_r_: 2, thickness 200 µm) on top of the wafer negligibly reduces the EF strength by about 3% [32]. Experimental results are shown in the photo presenting the varying dimensions of the fabricated lenses by applied voltage (V_in_) with a 0.5 kV increment. A V_in_ of 3.5 kV causes higher kinetic energy to the droplet than V_in_ of 1.5 kV; thus, the larger the V_in_, the larger the diameter (D) of lenses. Figure 5b shows the curvature of lenses. The larger radius of curvature (Rc) of the droplet is formed compared to one by the V_in_ of 1.5 kV. The results shown in the three images support the idea that the larger the V_in_, the smaller the static contact angles. It implies that the higher the static contact angle, the smaller the Rc and shorter the optical focal point, which means the graphene sensor channel can face a higher concentration of UV photon energy rather than a large Rc.

Figure 5c,d magnify the side and top view of the lenses with notations of the thickness of the lens center, Rc, and D of the lens. Herein, we highlight the effect of V_in_ on the dimensions of the lenses, but foremost, we confirmed that the diameters do not change (∆D D_0_^−1^) after the formation of the hydrophilic lenses on the hydrophobic SiO_2_. Additionally, the D (aperture) of the lens is secured during the printing process. Upon completion of solidification, the lens did not experience noticeable deformation as shown in Figure 5e. However, the D is enlarged and deformed if the sensor is not horizontally positioned before curing. In Figure 5f, to verify the optical functionality through the imaging lens mode, the deposited lens with the film is placed on a microruler that has black grid lines with an interval of 100 µm. As shown with the arrow, the fabricated PEI droplet magnifies the grid lines, verifying that it utilizes a refractive optical lens.

The overall dimensional quantification shown in Figure 5g–i presents how V_in_ can effectively modulate the dimension of the lenses, including the D, lens center thickness, and Rc, which affects the focusing of the UV ray. Figure 5g presents the dimensional variation of D and Rc as a function of V_in_, in which the higher V_in_ produces the larger D and Rc. Figure 5h shows the modification of thickness of the lens center as a function of V_in_, exhibiting that the higher V_in_ leads to the smaller thickness of the lens center. This consistently supports the tendency of D and Rc increasing as EF increases. The results also confirm that the spreading of droplets is primarily determined by EF-assisted kinetic energy [49]. Thus, controlling V_in_ in the printing process can vary the kinetic energy of the droplet, which effectively changes the dimensions of the lenses. Figure 5i shows the volume and change of the lateral surface area as a function of V_in_, whereas volume is calculated. The Rc and thickness of the lens center reveal that as V_in_ increases, the volume of the lenses shows negligible changes (<3.7%) because the droplet size is maintained through droplet printing, which is monitored through vision control in real time. Although the microcontroller can maintain the size of droplets, the volume of the printed droplet is slightly increased due to the inevitable pulling of the viscoelastic polymer ejected from the pipeline of the needle. Next, the plot shows that the larger the V_in_, the larger the lateral surface area.

### 3.2. Geometrical Analysis

**Figure 6:**
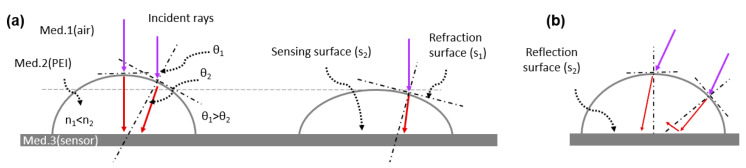
Geometrical analysis. (**a**) The relationship between incident angle, refraction angle, refraction surfaces, reflection, and mediums, according to Snell’s law. (**b**) Analysis of UV incidence at 30 degrees.

An optical principle to largely boost the UV sensitivity via the V_in_ is clarified in this section. The greater concentrated UV irradiance caused by the geometrical refraction of the lens gives the graphene sensor a higher sensing resolution. In general, a greater lateral surface area (wide aperture) absorbs and delivers more optical rays. Here, however, we provide a study of how the smaller lateral surface area aims for greater boosting. Figure 6a presents the relationship between the incident angle (θ_1_), refraction angle (θ_2_), refraction surfaces (S_1_), reflection surface (S_2_), and refractive index of mediums, according to Snell’s law (n_1_ sinθ_1_ = n_2_ sinθ_2_). A smaller Rc (left lens) leads to the larger difference between θ_1_ and θ_2_, resulting in the shorter focal point, in which the concentrated UV irradiance transmitted onto the sensing surface (S_2_) is greatly enhanced. On the other hand, when the same incident rays having the same θ_1_ pass through the thin droplet, the refraction enlarges the focal point, reducing the UV irradiance on the S_2_. This can demonstrate the relationship between material properties and sensing enhancement. A dome-shaped lens was printed using the property that the hydrophilic PEI solution does not spread on the sensor surface [41]. Conversely, if a hydrophobic solution is used, not only is it difficult to form a lens, but it also spreads widely and has the thin thickness of the lens, which reduces the optical focusing ability. In general, if the incident rays are propagated from a large angle as shown in Figure 6b, the S_2_ inevitably reflects rays due to its glossy surface. It was reported that the graphene-mounted SiO_2_ sensor reflects optical rays as a reflection surface, in which the amount of irradiance decreases [53].

### 3.3. Ray Optics Simulation

**Figure 7:**
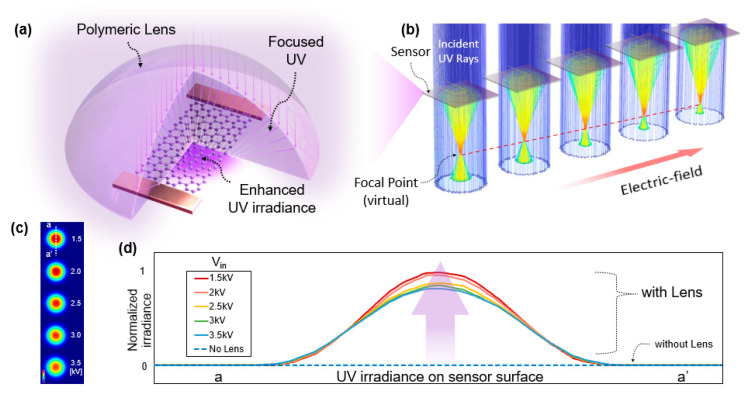
Optics simulation provides the effectiveness of ray refraction. (**a**) Focusing of UV rays onto the graphene channel. (**b**,**c**) EF-dependent UV concentration. The smaller applied voltage, V_in_, provides stronger UV irradiance on the sensor channel. (**d**) Normalized UV irradiance distribution in the cross-section of each lens, in which the UV irradiances with low V_in_ provide stronger UV.

Figure 7a provides a schematic of the optocatalytic boosting of the graphene-based photosensor. The one-step printed polymeric lens on the graphene-based sensor focuses UV rays onto the graphene channel. The graphene channel detects enhanced UV irradiance. Figure 7b shows the COMSOL optics simulation, presenting the modulation of UV exposure through the lenses where incident UV irradiance is concentrated similar to the virtual refracted rays. The dimensions of each lens are designed to be identical to the fabricated ones by V_in_ from 1.5 to 3.5 kV. According to the relationship between the focal point and dimension for the plano-convex droplet, the focal point is expressed as Rc (refractive index-1)^−1^. Figure 7c shows the simulation outcome on the output of UV irradiance on the S_2_. The result indicates that the lenses fabricated by the lower V_in_ deliver the higher UV irradiance (red color) with the smaller Rc (shorter focal point), allowing more enhanced UV energy on the sensing surface, i.e., graphene. Figure 7d plots irradiation curves of the a-a’ cross-section of each lens normalized by the irradiance without droplets (blue color, nonrefracted irradiance). The simulation result suggests that the curves of UV irradiance passing through the lenses have stronger maximum photon energy compared to the irradiance without the lens.

### 3.4. Verification: Improved Sensitivity

**Figure 8:**
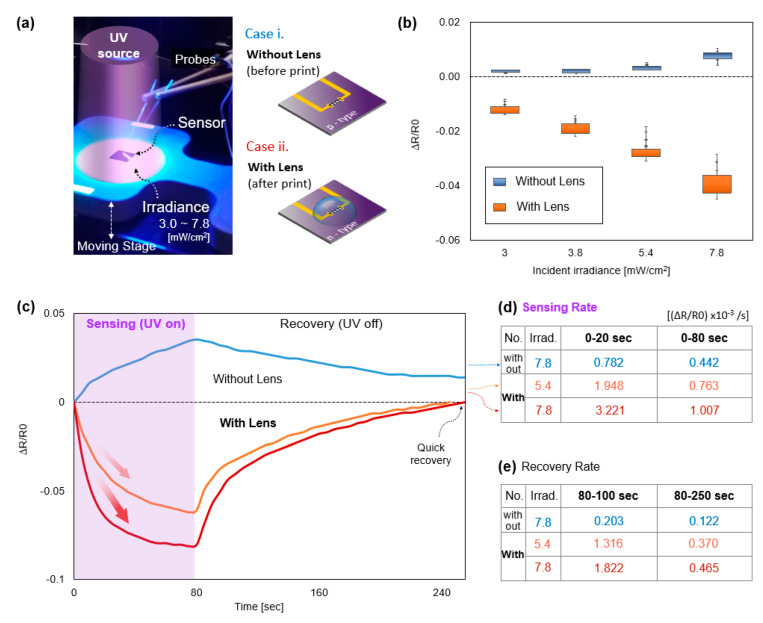
(**a**) A tabletop UV exposure and measurement system. Two types of sensors are tested: i. nonfunctionalized graphene sensor without lens (*p*-type, before droplet print), ii. enhanced sensor with a droplet (*n*-type, after droplet print). (**b**) Improved sensing of *n*-type graphene, in which the response of resistance is significantly changed. (**c**) Improved sensing rate and recovery rate in continuous UV-on/off mode. The concentrated UV provides intense photonic energy. Condensed molecules in the droplet promote the readsorption of electron-hole pairs. (**d**) Graphene with the lens exhibits a boosted sensing by about 330% at 0–15 s and by 220% at 0–45 s. (**e**) Lens provides a much faster recovery rate compared to the graphene without the lens by about 632% at 45–60 s and 540% at 45–180 s.

Figure 8a provides the experimental system for UV sensing. An exposure system includes a microscope, tungsten probes, and a Z-axis stage for adjusting the strength of the UV exposure. The irradiance, the intensity of incident UV reaching the sensor surface, is directly measured using the photometer. The ∆R is measured by a digital multimeter. Sensitivity, the ability to sense, is provided by the percentile resistance change (∆R R_0_^−1^). The UV irradiances of 3, 3.8, 5.4, and 7.8 mW cm^−2^ are set by adjusting the distance between the UV exposure module and the sensor surface. To fully demonstrate the feasibility and the functionality of the proposed method, we tested two cases of sensors: i. a nonfunctionalized graphene sensor without a lens (*p*-type, before droplet print), and ii. An enhanced sensor with a droplet (*n*-type, after droplet print). In this work, PEI-induced doping converts the graphene phase into an *n*-type [11], increasing the Fermi level higher than the Dirac point. The H_2_O molecules (50% in PEI solution) act as electron acceptors, thus the R of the graphene increases to the initial mode. Then, R decreases by photodesorption upon the UV exposure mode. Since the incident UV rays can penetrate the droplet and be refracted to the focal point, the concentrated UV provides intense photonic energy to the graphene, resulting in a significant increase in photodesorption. Figure 8b presents the measurement result of the photodesorption for each case at varying incident irradiances, in which sensors comparatively prove the effectiveness of optical refraction. The blue-marked box plot on top indicates that the nonfunctionalized pure sensor (type i, before droplet print) has low sensitivity and a nonmeasurable ∆R in the low UV irradiance of about 3 mW/cm^2^. On the other hand, the orange-marked graphene with a lens (type ii, after droplet print) has boosted ∆R. This implies that the droplet focuses the UV rays and concentrates photon energy, effectively enhancing photodesorption. The lens also magnifies ∆R in the low irradiance, which means that the suggested method practically utilizes a wide range of UV irradiance, such as a very low UV exposure environment.

Furthermore, rich H_2_O molecules in the droplet promote the readsorption of the initial resistance when the UV is off. As a result, the printed PEI lens contributes to the excitation and assembly of electron-hole pairs more apparently, in which the sensor performs boosting and recovery in ± ∆R. Figure 8c presents the continuous measurement of the sensing (UV-on mode) and recovery (UV-off mode) of the graphene-based UV photosensor with a lens and without a lens for 250 s. UV irradiance of 7.8 mW cm^−2^ is exposed for 80 s, and then the exposure is turned off to validate the recovery of the sensors. The sensitivity improvement is noticeable at the early stage of each mode. The graphene with the lens exhibits an improved sensing rate ((∆R/R_0_) × 10^−3^/s) by about 410% at 0–20 s and by 220% at 0–80 s in comparison to the graphene without the lens. Even if the incident irradiance is 5.4 mW, the sensing rate is 250% larger than the sensing without the lens. In addition, it was observed that the recovery rate of the graphene with a lens has an effective recovery rate compared to the graphene without the lens by about 890% at 80–100 s and 390% at 80–250 s after the UV irradiance of 7.8 mW cm^−2^ is exposed. The graphene with the lens still proves it has a fast recovery rate after the exposure to UV irradiance of 5.4 mW cm^−2^ compared to the one without the lens under the exposure to UV irradiance of 7.8 mW cm^−2^. These results conclude that the existence of a lens apparently boosts the sensitivity and recovery rate of the graphene-based UV photosensor.

**Figure 9:**
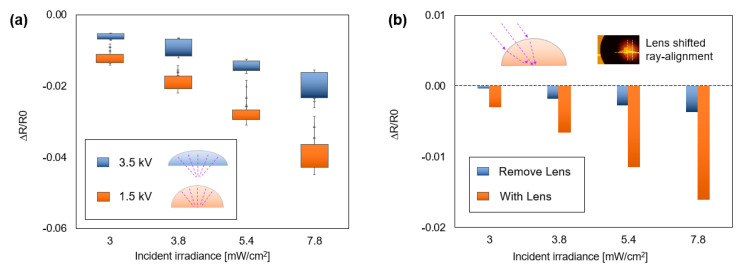
(**a**) Experimental result on the sensing performance of lenses. Printing with lower V_in_ is beneficial for boosting photodesorption. (**b**) The droplet can boost photosensing where UV propagates from various angles.

Figure 9a shows a variation of concentration according to Rc produced by V_in_, which shows selectivity of sensitivity. In accordance with the simulation result in Section 3.3, the droplet with the smaller Rc printed at 1.5 kV (orange) exhibits a higher ∆R at every UV incident irradiance level compared to the one with the larger Rc printed at 3.5 kV (blue). Therefore, the results satisfy the theoretical strategy and simulation that printing with a low V_in_ is beneficial for promoting photodesorption. In addition, Figure 9b shows the sensing with UV incidence at 30 degrees relative to the vertical axis. This measurement provides an additional advantage to the 3D convex lens that can boost photosensing where UV propagates from various angles. In Section 3.3, we noted that if the incident rays have a large-angle θ_2_, the S_2_ can reflect rays [53], diminishing the irradiance and the sensing response. Therefore, overcoming the reflection contributes to improving higher ∆R. For the test, the coordination of printing is shifted correctly according to the ray alignment, as shown in the subset image. The result shows that the lens improves sensitivity by about 450%. Next, UV sensing with the n-doping-only sensor (after droplet removal) at 30 degrees shows a tendency to have lower sensitivity than vertical UV exposure, which means that the reflection induces a small ∆R. Lastly, the lens grants that enhanced UV sensing overcomes reflection. Unlike the general absorption in planar graphene, the S_1_ on the nonplanar boundary of a three-dimensional convex lens provides diminished reflection, resulting in boosted ∆R. However, to leverage this function, verification using various incident angles and wavelengths is required.

## 4. Conclusions

This study provides facile fabrication and enhancement of atomically thin material-based optoelectronic devices through electric-field-assisted polymer printing. The printed droplet not only dramatically enhances the sensitivity, but also improves the recovery rate. The reliability of the printing method and boosting mechanism are proven by a series of experiments. The proposed method promises a single-step printing of tunable polymeric lenses by leveraging a number of advantages, including a low material cost, simple process, and controllability. By implementing individually controllable polymeric lenses, optical focusing and catalytic doping, together, successfully boost UV photodetection. To support this presentation, we can expect more experimental evidence of photo readsorption and to shorten the curing time by using transparent UV-curing polymers.

Note that the two-dimensional materials in their original planar form do not utilize the full advantage of the multi-dimensional physical nature. An embedding three-dimensional polymeric optostructure can provide an extensive platform for the enhancement of performance and applications. This method is envisioned to broaden its applications through scaling up by incorporating multiple needles as well as utilizing a wide range of functional polymers and encapsulations for varying purposes. This facile method is expected to be applicable to a wide range of fields, including research, prototyping, optical and energy devices, and hybrid manufacturing processes.

## Data Availability

The data presented in this study are available on request from the corresponding author.
